# Regulation of Precise DNA Repair by Nuclear Actin Polymerization: A Chance for Improving Gene Therapy?

**DOI:** 10.3390/cells13131093

**Published:** 2024-06-24

**Authors:** Xiubin He, Cord Brakebusch

**Affiliations:** Biotech Research and Innovation Centre (BRIC), University of Copenhagen, Ole Maaløes Vej 5, 2200 Copenhagen, Denmark; xiubin.he@bric.ku.dk

**Keywords:** nuclear actin, actin dynamics, actin polymerization, double-strand DNA breaks, DNA repair, DNA damage response, homologous recombination, chromatin remodeling, genome integrity

## Abstract

Although more difficult to detect than in the cytoplasm, it is now clear that actin polymerization occurs in the nucleus and that it plays a role in the specific processes of the nucleus such as transcription, replication, and DNA repair. A number of studies suggest that nuclear actin polymerization is promoting precise DNA repair by homologous recombination, which could potentially be of help for precise genome editing and gene therapy. This review summarizes the findings and describes the challenges and chances in the field.

## 1. Introduction

Rare diseases are diseases with a frequency of less than 0.05%. More than 7000 rare diseases are known to be affecting about 350 million people worldwide [1]. Most rare diseases are caused by mutations, and gene therapy is therefore the only curative treatment option for these patients. CRISPR gene editing is an exciting possibility for therapeutic repair of defective genes in rare diseases [2,3]. Here, a complex of a Cas9 protein with a short guide RNA function as a genomic “scissor”, which precisely cuts the genome at a single place close to the mutation, introducing a double-strand DNA break (DSB). In the presence of a repair template, the defective mutation is repaired precisely without leaving any traces in the genome. In the absence of a repair template and in competition with precise gene editing, an error-prone repair takes place, which introduces further mutations at the DSB. To increase the efficiency of gene therapy and reduce costs, a large amount of research is currently trying to increase the efficiency of precise repair during CRISPR gene editing. Nuclear actin polymerization is a candidate pathway to promote precise gene editing.

Actin is expressed in all cells and contributes by polymerization to the cytoskeleton and cell morphology. Regulation of the actin cytoskeleton is crucial for cell migration and adhesion by controlling membrane protrusions and cell contraction [4,5]. It also plays a role in endocytosis and intracellular transport of vesicles and organelles [6]. In recent years, it has been revealed that actin polymerization can also occur in the nucleus, mostly in response to stress such as serum stimulation or irradiation. This was reported to influence transcription, translation, replication, and DNA repair [7,8]. With respect to DNA repair, nuclear actin polymerization was often linked to the promotion of precise repair by homologous recombination (HR) [9]. In this review, we describe the basics of actin polymerization and the repair of DSBs, and we present and discuss the current knowledge of how nuclear actin polymerization and DNA repair might be linked.

## 2. Regulation of Actin Polymerization

**Figure 1:**
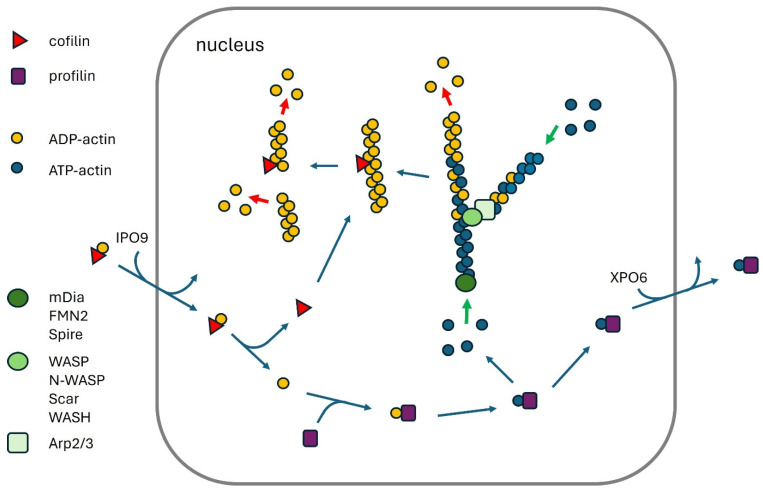
Regulation of nuclear actin polymerization. ADP-actin-cofilin is imported into the nucleus by IPO9, while profilin exports ATP-actin to the cytoplasm with the help of XPO6. Profilin binding to actin promotes the exchange of ADP for ATP, which facilitates actin polymerization. Formin proteins (mDia, FMN2) contribute to linear F-actin formation, which is accelerated by Spire. Arp2/3, together with Wasp family proteins, mediates branched F-actin formation. Cofilin facilitates actin depolymerization by severing ADP-bound F-actin.

Actin exists in two forms: the monomeric actin (G-actin) and the filamentous actin (F-actin). These forms convert to each other in a dynamic equilibrium that is controlled by a large number of actin-binding proteins (ABPs) (Figure 1) [8]. Above a critical concentration, G-actin can polymerize spontaneously to F-actin, but G-actin-binding molecules such as profilin reduce the concentration of free G-actin in the cell below the critical level, preventing uncontrolled polymerization. Profilin also promotes the exchange of ADP bound to actin by ATP, which is a key step for actin polymerization (Figure 1). At low concentrations of free actin, actin polymerization is triggered by actin nucleation-promoting factors (NPFs). Formins such as mDia and Formin 2 (FMN2) promote the linear polymerization of actin filaments, whereas the Arp2/3 complex, together with Wasp family proteins, initiates the growth of branched actin networks (Figure 1) [10,11]. A third group of NPFs are tandem-monomer-binding proteins such as Spire. Force generated by actin polymerization can mediate membrane protrusions or intracellular movements of bacteria in mammalian cells [4]. Crosslinking proteins like filamin, fimbrin, and α-actinin organize F-actin into networks and bundles, providing structural integrity to the cytoskeleton [12,13,14]. Within the actin filaments, actin-bound ATP is hydrolyzed to ADP, which allows binding and severing of the filaments by cofilin and stimulates depolymerization at the pointed end of the filaments (Figure 1) [5]. Barbed-end capping proteins like CapZ and gelsolin bind to the fast-growing, barbed end of actin filaments, inhibiting elongation, while the pointed-end capping protein tropomodulin can inhibit depolymerization. Myosins can “walk” on F-actin in an ATP-dependent manner, which is used for cell contraction or for the intracellular transport of vesicles and organelles [15]. Finally, many signaling pathways regulate the activity of ABPs by protein–protein interaction, post-translational modification, or by regulating their expression [15].

## 3. Actin Polymerization in the Nucleus

Monomeric and filamentous actin are not only found in the cytoplasm but also in the nucleus. In addition, many ABPs are present in the nucleus, suggesting that regulated actin polymerization in the nucleus might be of physiological importance for nuclear processes [8]. Although monomeric, unbound actin, based on its small size of 42 kD, should be able to freely diffuse to the nucleus, it uses energy-dependent transport processes to quickly move in and out of the nucleus. Import of G-actin to the nucleus is mediated by unphosphorylated cofilin binding to preferentially ADP-bound G-actin and importin 9 (IPO9) (Figure 1) [16,17]. Phosphorylation of cofilin will prevent its binding to actin and, consequently, nuclear import. Regulation of cofilin phosphorylation by LIM kinase and slingshot phosphatase appears therefore as a major physiological pathway to modulate the nuclear import of G-actin [18,19]. Indeed, phosphorylation of cofilin during mitotic exit corresponded to the formation of nuclear F-actin [20], and cofilin regulators were identified in a genome-wide screen for inhibitors of nuclear actin localization and polymerization [21].

Export from the nucleus requires binding of G-actin to profilin and exportin 6 (XPO6) [22]. Inhibition of the nuclear export of actin by knockdown (KD) of XPO6 increases nuclear G-actin levels and promotes nuclear F-actin formation [23,24].

Interestingly, unphosphorylated cofilin not only promotes the nuclear import of G-actin but also severs F-actin [25]. Thus, active cofilin in the cytoplasm will promote both depolymerization of F-actin in the cytoplasm and transport of G-actin to the nucleus (Figure 1), suggesting that nuclear G-actin levels are increasing when cytoplasmic actin depolymerization is high. In addition, active cofilin mediating nuclear transport of G-actin might promote the severing and depolymerization of ADP-bound F-actin in the nucleus, resulting in a short lifetime of nuclear actin filaments induced by cofilin dephosphorylation. On the other hand, profilin not only promotes the nuclear export of actin but also facilitates the polymerization of ATP-actin by binding to NPFs [5]. These contrasting relationships might suggest that actin polymerization is less likely in the nucleus than in the cytoplasm, and that normally, nuclear F-actin in unstressed mammalian cells is much more difficult to detect than cytoplasmic F-actin. Whether the relatively low amounts of nuclear actin polymerization are due to evolutionary pressure, trying to avoid toxically high levels of nuclear actin polymerization, is unclear.

## 4. Repair Mechanisms of Double-Strand DNA Breaks

**Figure 2:**
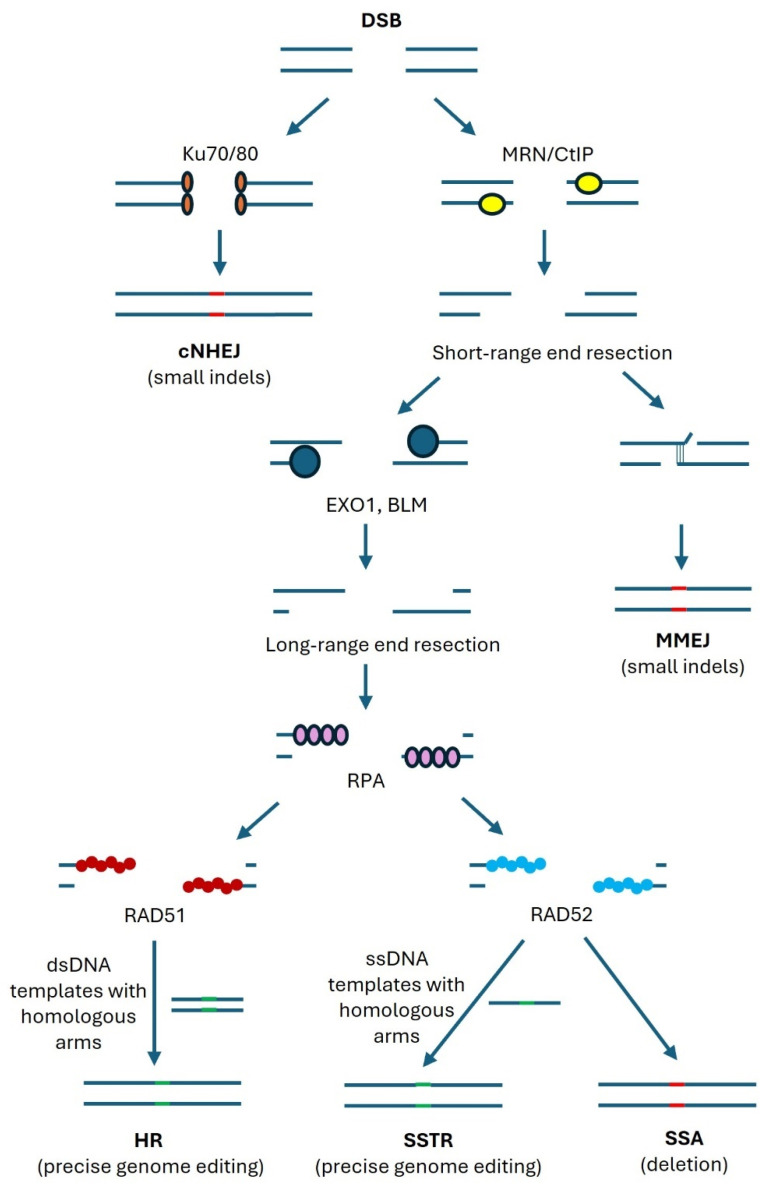
Repair pathways of DSB. In the cNHEJ pathway, Ku70/80 proteins bind to DSBs, which results in small deletions and insertions. When the MRN-CtIP complex binds to DSBs, short-range end resection occurs and ssDNA overhangs are generated, which can lead to the MMEJ pathway. DSBs can further enter long-range end resection mediated by proteins such as EXO1 or BLM, where the long ssDNA overhangs are bound by RPA. RPA is then replaced by RAD51 or RAD52, which direct repair to HR or SSA and SSTR, respectively. Indels are indicated in red, and precise genome editing is indicated in green.

DSBs can be repaired by error-prone mechanisms, including classical nonhomologous end joining (cNHEJ) and microhomology-mediated end joining (MMEJ), or by precise homologous recombination (HR) (Figure 2).

cNHEJ is started by the binding of Ku70/Ku80 dimers to the DSBs, which triggers the association with other repair-associated proteins [26]. The free DNA ends are either ligated directly or after some processing by nucleases or polymerases, which often results in insertions or deletions (indels).

On the other hand, binding of CtIP and the MRE11-Rad50-NBS1 (MRN) complex, which contains the nuclease MRE11, to DSBs is initiating end resection, which is the generation of single-stranded overhangs on both sides of the DSBs [27]. This process occurs only during the S and G2 phases [28,29], while cNHEJ is possible during all phases of the cell cycle [26].

If the overhangs are about 20 bps, a short stretch of complementary base-pairing can associate the opposite sides of the break, which is then sealed by the combined action of nucleases and polymerases [30]. This repair is called MMEJ and results in a short deletion of DNA.

Longer end resections, mediated by helicases and nucleases such as EXO1 and BLM, will enable the binding of replication protein A (RPA) to the single strand ends, which is later replaced by RAD52 or RAD51 [31,32,33]. In the absence of a template, complementary base pairing of RAD52-bound stretches longer than 20 bp can trigger deletion-causing DNA repair. This repair pathway is called single-strand annealing (SSA). In the presence of a single-stranded DNA (ssDNA) template with homologous sequences on both sides of the DSBs, precise genome editing can be performed (single-stranded template repair, SSTR). RAD51-bound single strands can undergo HR with the help of double-stranded DNA (dsDNA) templates with homologous sequences on both sides of the DSB. Templates could be sister chromatids or exogenously provided DNA.

Precise genome editing via HR is a hope for patients suffering from rare diseases that are caused by mutations. Genome editing by CRISPR/Cas9 introduces a DSB close to the mutation, which is then repaired with the help of a template. Unfortunately, error-prone repairs by cNHEJ and MMEJ are mostly outcompeting the precise edits by SSTR and HR, resulting in a low percentage of correctly repaired genes. For the clinical application of CRISPR genome editing, it is therefore essential to maximize the percentage of cells that are repaired by precise DNA repair. Mechanisms that can contribute to this goal should be investigated for their potential use in gene therapy, and nuclear actin polymerization is a candidate pathway for promoting HR.

## 5. Methodological Challenges

Investigating the role of nuclear actin polymerization in DSB repair faces several methodological problems.

Firstly, the effect of nuclear actin polymerization might be dependent on the method applied to induce DSBs. DSBs can be induced by different methods. Often used are irradiation or chemical reagents [34,35] that induce multiple DSBs but also have side effects such as the production of reactive oxygen species [36]. In addition, different DSB-inducing agents have different preferences with respect to the target site [37]. A more subtle method for DSB induction is the expression of sequence-specific nucleases that cut the genome at single or multiple DSBs [38,39]. Yet, only in the latter case will it be possible to detect effects based on the clustering of multiple DSBs.

Secondly, the method used for detection of nuclear actin polymerization might be either not nucleus-specific or include the risk of directly affecting actin polymerization. A common way to detect F-actin in fixed cells is by staining them with fluorescently labeled phalloidin [20]. However, the high amount of cytoplasmic F-actin makes it, in most cases, difficult to clearly identify potential nuclear F-actin. In corresponding wide-field images, stress fibers can appear to extend from the cytoplasm into the nuclear area, although they are in fact located above the nucleus. In confocal or super-resolution microscopy, these false positive nuclear stress fibers appear significantly weaker in the apparent nuclear area and can be identified as extranuclear in 3D stack analyses.

Detection of nuclear F-actin by transfection of cells with fluorescent protein probes binding to F-actin and targeted to the nucleus by a nuclear localization sequence (NLS) has two advantages: They largely avoid the detection of cytoplasmic F-actin, and they allow the monitoring of nuclear F-actin in living cells. Some of these probes were shown to promote the non-physiological formation of F-actin, while others appeared to be neutral [40]. However, depending on the expression level and the cellular system used, artifact formation can never be completely excluded [41]. Actin chromobodies are intracellularly expressed fluorescent proteins that bind to actin via an antibody-derived protein domain. Cobb et al. reported that following expression of actin chromobodies in U2OS cells, about 20% of the cells showed clearly visible nuclear nodules or fibrils, which in some cases were thick, long, and bended [42]. Palumbieri et al. stably expressed a FLAG-tagged NLS-actin fusion protein at low levels in U2OS cells, which was detected after fixation by immunofluoresent staining for the FLAG tag [43]. With this system, they detected nuclear foci and fibrils in about 30% of the untreated cells. Such structures have not been described before in phalloidin-stained, untransfected U2OS cells. One possible explanation for this discrepancy would be that actin chromobodies and FLAG-tagged actin might be much more sensitive in detecting nuclear F-actin than phalloidin. Another explanation would be that these probes stabilize F-actin structures and, by this mechanism, increase nuclear F-actin.

In MCF7 cells, transient expression of actin chromobodies detected long and bended nuclear F-actin only following inhibition of p53 [44]. Importantly, these F-actin structures could easily be detected by phalloidin staining, but only in cells transfected with actin chromobodies. Similarly, nLifeact-GFP expression resulted in phalloidin-stainable, thick nuclear actin filaments, while cells not expressing the probe showed no nuclear F-actin detectable by phalloidin. These data suggest that actin-binding probes expressed in living cells indeed have the potential to induce nuclear F-actin.

As a final methodological challenge, it is difficult to specifically interfere with nuclear actin polymerization. Nuclear and cytoplasmic actin pools are connected in a dynamic equilibrium, and nuclear–cytoplasmic transport is regulated by the amounts of actin transport molecules and by their post-translational modifications [16]. Molecules regulating actin polymerization are often present both in the cytoplasm and the nucleus. Targeting molecules in the nucleus with an NLS sequence might lead to unphysiologically high levels of this protein in the nucleus. Targeting a non-polymerizable actin mutant in the nucleus reduces nuclear actin polymerization but might have effects on nuclear proteins that bind G-actin. Inhibitors, such as KD or knockout (KO), will efficiently work on the nuclear protein but also on cytoplasmic proteins, which indirectly might affect DNA repair.

Torii et al. explored the effect of the overexpression of nuclear-targeted and GFP-coupled wild-type and S14C actin, an actin polymerization-promoting mutant [44]. With wild-type actin, inhibition of p53 and treatment with doxorubicin promoted the formation of thin nuclear actin filaments not detectable by phalloidin. In contrast, S14C actin resulted in actin nodules with very thin, short fibrillar extensions, all of which could be stained by phalloidin. While the molecular reason for these differences is not clear, they indicate that results obtained with NLS-actin-GFP fusions as well as with actin mutants need to be interpreted very carefully.

## 6. Nuclear Actin Polymerization Promotes DNA Repair by HR

Several studies have demonstrated that actin and ABPs bind to DSBs and promote actin polymerization and clustering of DNA repair foci, which are membrane-less condensates that form around sites of DNA damage [45]. Importantly, nuclear actin polymerization is often correlated with increased precise genome editing.

The first evidence of nuclear actin potentially being involved in DSB repair came from an F-actin disruption experiment in human MCF-7 breast cancer cells [46]. Shin et al. uncovered that protein levels of phosphorylated histone H2AX (γH2AX), a marker of DSB, were dramatically increased after latrunculin B (LatB)-induced actin disruption [46]. Subsequently, Andrin et al. confirmed a direct association between polymerized actin and DSB repair proteins, including Ku70, Ku80, Mre11, and Rad51, through in vitro F-actin co-precipitation [47]. Of note, these proteins include repair proteins required for cNHEJ as well as for resection and HR. In addition, the authors showed by different assays that inhibition of actin polymerization inhibits the repair of irradiation-induced DSBs, particularly cNHEJ [47]. However, neither of the above evidence could prove a direct interaction between nuclear F-actin and DNA repair proteins, as the binding might be mediated by ABPs. Since expression of an NLS-tagged non-polymerizable actin mutant (G13R) reduced retention of Ku80-GFP at laser micro-irradiation-induced DNA defects similar to treatment with the actin polymerization inhibitor cytochalasin D [47], cytoplasmic actin polymerization appeared not to be involved in the observed effects.

**Figure 3:**
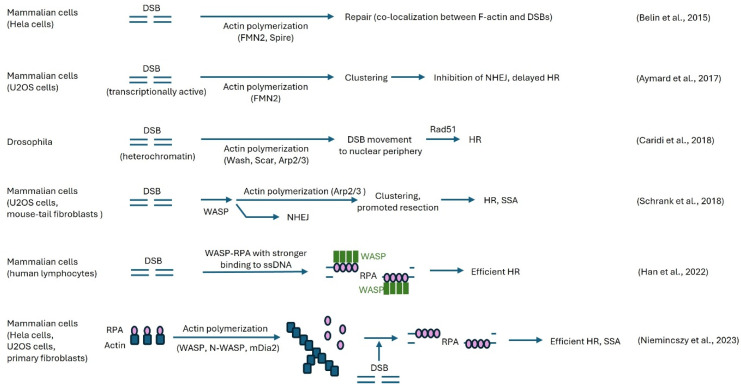
Nuclear actin polymerization-dependent regulation of DNA repair. Presented are different studies indicating the involvement of nuclear actin polymerization in the repair of DSBs with the corresponding suggested molecular mechanisms. The clustering and movement of DSBs were reported to depend in some models on FMN2 [42,44], but in other systems on Arp2/3-dependent actin polymerization [45,46]. WASP was found to release RPA bound to G-actin [48], but also to bind to DSBs and facilitate end resection by nuclear F-actin formation [46]. Independent of actin polymerization, WASP binding to RPA was demonstrated to increase RPA binding to ssDNA [47].

Later, Belin et al. detected an increased amount of both long and short actin filaments in the nucleus induced by DNA-damaging agents such as methyl-methanesulfonate (MMS) using phalloidin staining and live-cell actin probes in Hela cells [23]. The experiments showed that DNA damage-induced nuclear actin assembly requires the nucleation factors FMN2 and Spire-1/Spire-2 but not mDia1/2, which earlier were shown to trigger nuclear actin polymerization (Figure 3) [23,48]. Depletion of either FMN2 or IPO9 increased the number of DSBs detected by 53BP1 or γH2AX as markers [23]. The authors considered this observation to be inefficient DSB clearance in the absence of nuclear actin filaments, but it cannot be excluded that the clustering of DSBs contributes to the decrease in DSB foci. The mechanism by which nuclear F-actin DNA promotes DNA repair was not studied.

Utilizing a high-throughput chromosome conformation capture assay (capture Hi-C), Aymard et al. revealed that DSBs at transcriptionally active genes cluster in the G1 cell cycle phase, which coincides with delayed repair [49]. Clustering depended on the MRN complex, the actin nucleation promoting formin FMN2, and the linker of the nuclear and cytoplasmic skeleton (LINC) complex, which connects structural proteins close to the internal nuclear membrane with the actin, microtubule, and intermediate filament networks of the cytoplasm. The researchers proposed that DSB clustering may help to sequester DSBs and prevent error-prone cNHEJ repair in G1, enabling delayed but precise DSB repair by HR in G2 [49]. This study suggested that nuclear actin polymerization is required in DSB clustering-driven repair by HR (Figure 3). However, the specific mechanism of how actin polymerization triggers DSB clustering and whether indeed nuclear actin polymerization is involved were not tested.

In *Drosophila*, repair of DSBs in heterochromatic regions by HR involves the DSB movement to the nuclear periphery before Rad51 recruitment, probably to avoid aberrant recombination with other DNA sequences than the sister chromosome [50]. Caridi et al. demonstrated that this DSB movement is dependent on Arp2/3-mediated nuclear actin polymerization and independent of the actin nucleators Spire and mDia (Figure 3) [50]. Furthermore, siRNA experiments demonstrated that Arp2/3 is activated by Wash or Scar but not by WASP or Whamy. Following irradiation, Arp2/3 co-precipitated with the heterochromatin repair complex Smc5/6, suggesting a close coupling of DSB repair with actin polymerization. Heterochromatic DSB movement also required myosin and the myosin activator Unc45. In contrast to heterochromatic DSBs, euchromatic DSBs rarely moved to the periphery, and undamaged heterochromatic DNA hardly moved at all. Interestingly, Arp2/3 was not important for clustering of early repair foci in heterochromatin, while it was essential for euchromatic early repair foci clustering.

Immunofluorescence staining of endogenously expressed WASP, an Arp2/3 stimulating NPF, revealed its localization at all DSBs in U2OS sarcoma cells and mouse-tail fibroblasts (MTFs) [51]. However, only at DSBs undergoing resection in the G2 cell cycle phase did Arp2/3 dependent actin polymerization take place, resulting in DSB clustering. These clustered DSBs were positive for RPA, RAD51, or RAD52, suggesting that clustering might affect DNA repair by HR and SSA (Figure 3). Indeed, inhibitors of Arp2/3-dependent actin polymerization or of WASP reduced HR and SSA, as shown by I-SceI assays, where restriction enzyme-induced DSBs are repaired in a template-dependent or independent manner [51]. Neither FMN2 nor other formins such as mDia1 and mDia2 were involved in this effect, as shown by KD and inhibitors. Interestingly, MMEJ or cNHEJ, neither of which require substantial resection, were not impaired by inhibiting WASP or Arp2/3, implying a resection-promoting role of actin polymerization in HR repair. The authors presented a model where resection enhances DSB movement, which, in a positive feedback-loop, increases resection [51]. How WASP is activated after recruitment to DSBs only at DSBs undergoing resection was not clarified.

Recently, Lamm et al. showed that aphidicolin-induced replication stress stimulated nuclear actin polymerization involving IQGAP1, WASP, Arp2/3, and inhibition of cofilin by phosphorylation [52]. Nuclear actin polymerization was found to contribute to replication fork repair. Here, myosin II was required for the movement of the replication foci. This suggests that the machinery required for the movement of DSBs and replication foci is partially overlapping.

A different role for WASP was suggested by Han et al. [53]. They observed that WASP interacts with RPA, which increases its interaction with ssDNA (Figure 3). WASP KO leads to less RPA binding to ssDNA and increased γH2AX staining. This could indicate the accumulation of unrepaired DSBs due to insufficient HR and SSA, which are both dependent on RPA. In yeast, SSA-induced deletion was not significantly influenced by loss of the WASP orthologue Las17, but DSB repair by SSA was even improved by Las17 deficiency. WASP-deficient B and T cells showed differential cell cycle changes to genotoxic stress dependent on the genotoxin, which is of interest as resection-dependent repair only occurs in S/G2 [53].

A completely different way of regulating RPA binding to ssDNA was proposed by Nieminuszczy and colleagues [54]. They showed that RPA can bind to actin and that this interaction prevents RPA binding to ssDNA. Actin polymerization releases RPA from actin, allowing RPA to bind to ssDNA and promote resection dependent DNA repair (HR and SSA) (Figure 3). KD of the actin nucleators WASP, N-WASP, or mDia1 all showed a similar increase in DSBs in the S/G2 phase identified by RBP1 foci following HU treatment. In this case, no direct interaction of the actin polymerization machinery with the DSB appeared necessary.

A principally similar indirect type of regulation was earlier described for the MAL/MRTF transcription factor. In the cytoplasm, MAL/MRTF is bound to G-actin [55]. Actin polymerization releases MAL/MRTF, which can then travel to the nucleus, where it stimulates transcription in collaboration with the SRF transcription factor [56]. Potentially, nuclear actin polymerization might influence the nuclear activity of MAL/MRTF [57]. Also, for the regulation of MAL/MRTF signaling, the exact type of NPF triggering actin polymerization is not relevant.

Related to this principle of regulation, nuclear RNA polymerases and several chromatin-modifying complexes, such as INO80 or TIP60, were shown to bind to monomeric actin, which appears to be important for the stability and function of these complexes [58,59,60]. If nuclear actin polymerization reduces the availability of G-actin, the stability of these chromatin-modifying complexes might be decreased. Importantly, these actin monomer-binding complexes might affect DNA repair, as was shown, for example, for TIP60 and INO80 [60,61,62,63,64,65].

Taken together, most of these studies indicate that nuclear actin polymerization promotes DSB repair by HR and SSA by facilitating resection. The exact molecular mechanisms underlying it, however, are not entirely clear. Several, maybe parallel, mechanisms were suggested involving different types of actin NPFs. To further investigate this process, it will be helpful to induce DSBs by methods with fewer side effects on other cellular processes than irradiation or chemical reagents. Secondly, the use of gene KO in the cell lines of interest could complement the published siRNA and small molecule inhibitor studies, which have a higher chance for partial as well as off-target effects. Rescue of the KO models by re-expression of the wild-type molecule would safely confirm that the observed effect is indeed related to the mutated gene. Thirdly, performing the rescue with molecules fused to nuclear export sequences (NES) or NLS and directing the proteins to the cytoplasm or nucleus, respectively, might help to distinguish between cytoplasmic and nuclear functions.

## 7. DSB Movement and Repair

The molecular details of how DSB movement or clustering support DNA repair by HR or SSA are not clear. One option would be that DSBs are moved from a region with a low likelihood of HR to a region with a high likelihood. Similarly, DSB clustering might increase the local concentration of HR-related proteins, facilitating recombination. On the other hand, increased DSB concentration could also increase the competition for HR-related proteins.

Several lines of evidence have suggested chromatin mobility changes in response to DSBs [49,66,67,68,69,70]. The pattern of chromatin mobility after DNA damage could be different depending on the DSB location, repair sites, and time [66,71,72]. Chromatin motions proximal to DSB sites retain higher mobility, whereas the motions of chromatin distal to DSBs are globally reduced [66]. In yeast and human cells, the motion of damaged DNA requires HR machinery [49,73], but it is not clear how DSB clusters direct to repair-conducive sites, how they interact with HR regulators, and in which role nuclear actin is involved. Another open question is how the directionality of the movement is controlled.

However, the clustering and spatial compartmentalization of DSBs triggered by WASP-Arp2/3-dependent nuclear actin polymerization also increase the risk of chromosomal translocations [74,75,76]. A study found that the location of DSBs proximal or distal to the centromere exhibits different mechanisms in DNA repair [71]. DSB mobility near the centromere relied on γH2AX, independently of the Rad9-dependent checkpoint and the Rad51 nucleofilament. The movement of sub-telomeric distal DSBs, on the other hand, contingent on the presence of a homologous sequence close to the centromere, required both Rad9 and the Rad51 nucleofilament essential for HR [71,77].

## 8. Is Nuclear Actin Polymerization Required for Genome Maintenance?

Defects in DNA repair caused by the alterations of nuclear actin polymerization could promote the frequency of mutations and contribute to the development of cancer.

Wiskott–Aldrich syndrome patients lack a functional WASP-encoding gene and suffer from severe immunosuppression and a predisposition to non-Hodgkin lymphoma and leukemia [78]. Lymphocytes from WAS patients displayed defective resection, implying impaired DNA repair by HR [51]. Whether this predisposition to leukemia and lymphoma is caused by defective HR repair, however, has not been shown yet. Since all nuclear actin polymerization regulators identified also have cytoplasmic functions, mutations in these genes will most likely influence both nuclear and cytoplasmic functions, making it difficult to prove a correlation between defective nuclear function and cancer development.

## 9. Can Facilitated Actin Polymerization Promote Genome Editing by HR?

While the inhibition of actin polymerization was shown by many groups to interfere with DSB repair by HR, it is still not clear whether it will be possible to facilitate nuclear actin polymerization in a way that promotes DSB repair by HR or SSTR, which would be of interest for ex vivo gene therapy of stem cells. If the release of RPA from monomeric actin is a rate-limiting step, all treatments that elevate nuclear actin polymerization would be supportive. However, transient knockdown of the nuclear actin exporter XPO6 in HeLa cells promoted nuclear actin polymerization but did not alter the frequency of MMS-induced DSBs as detected by 53BP1 foci [47].

The movement of DSBs was shown by several groups to occur exclusively at DSBs undergoing HR [50,51]. It would be interesting to test whether it is possible to increase the fraction of DSB undergoing movement by altering the regulation of nuclear actin polymerization and whether this would increase the fraction of DSBs that are repaired by HR.

Irradiation was reported to cause nuclear activation of RhoA [79], a Rho GTPase that in the cytoplasm induces actin polymerization via the mDia formins and via activation of myosin-dependent contraction. It was reported that RhoA inhibition increased the number of DSBs by irradiation and impaired the NHEJ pathway in glioma cells in a p53-dependent way, while HR was only affected in p53-mutant cells [80]. However, the effect of nuclear RhoA activation or inhibition on HR has not yet been investigated.

When in a closed conformation, WASP is neither able to bind to Arp2/3 nor to induce actin polymerization. The binding of GTP-bound Cdc42 to WASP induces a conformational shift and enables actin polymerization. Whether this WASP activation scheme could be integrated with the proposed binding of WASP to all DSBs, while Arp2/3 is binding only to those DSBs undergoing resection, needs to be studied. In any case, the regulation of the actin polymerization activity of nuclear WASP will be of interest.

Cofilin controls the nuclear import of actin as well as the severing of nuclear F-actin. Cofilin dephosphorylation, promoting its binding to ADP-actin, was reported to transiently increase nuclear F-actin [20]. Whether this increase affects DSB repair was not tested. On the other hand, siRNA against cofilin promoted nuclear F-actin during mitotic exit [20], while it prevented nuclear actin accumulation induced by XPO6 siRNA [16]. This suggests that the regulation of cofilin needs to be carefully regulated to obtain the desired result of more nuclear F-actin due to reduced severing.

## 10. Conclusions

The current data do not identify strong candidates for actin polymerization regulating genes that could be transiently tuned to promote precise genome editing. However, an improved molecular understanding of how nuclear actin polymerization is controlled might provide such targets. For future experiments, phalloidin staining appears to be the gold standard for monitoring nuclear F-actin. If live cell actin probes are used, it should always be controlled whether their expression affects nuclear F-actin as detectable by phalloidin staining. If nuclear actin polymerization is manipulated by inhibition, overexpression, or knockout of actin regulators controlling, for example, nuclear transport, polymerization, or severing of F-actin, it needs to be demonstrated that these actin regulators are altered in activity or expression under physiological circumstances. Moreover, under physiological conditions, changes in actin regulators should correspond to changes in nuclear F-actin. Distinguishing the effects of nuclear actin polymerization from effects on actin monomer-binding chromatin modifier complexes will remain a challenge. Perhaps testing the consequences of nuclear actin polymerization on DSB repair in cell lines with KO of one or several genes of chromatin modifier complexes could be an option. DSB induction by CRISPR will be a subtle method to trigger DNA repair, avoiding the unwanted side effects associated with irradiation or chemical carcinogens, which indirectly might affect DSB repair. Finally, testing the consequences of altered actin polymerization directly on the HDR and NHEJ efficiency of CRISPR gene editing will increase the chances for novel findings that improve the efficiency of gene therapy for rare diseases.
